# Transcription Factors in Plant Gene Expression Regulation

**DOI:** 10.3390/ijms26209955

**Published:** 2025-10-13

**Authors:** Piotr Szymczyk

**Affiliations:** Department of Biology and Pharmaceutical Botany, Medical University of Łódź, Muszyńskiego 1, 90-151 Łódz, Poland; piotr.szymczyk@umed.lodz.pl

Gene expression is a fundamental element in the process of genetic information flow [1]. The elemental character of DNA transcription cannot be downgraded in further stages of gene information execution such as splicing, RNA transport, translation, or protein folding, and post-translational modification; ultimately, proteins with precisely defined functions maintain the occurrence of a particular phenotype [2]. The localization of actively transcribed genes usually corresponds with the open chromatin state lacking nucleosome organization and more prone to nuclease digestion [3,4]. These areas, known as DNAase hypersensitive sites (DHS) are found in promoters, enhancers, and silencers in animal and plant genomes [4]. In the *Arabidopsis thaliana* genome, approximately 38,290 and 41,193 DHS sites in leaf and flower tissues were observed, respectively [5].

Information on gene expression in the context of spatio-temporal and biotic–abiotic stress response is mostly derived from the 5′ UTR regulatory sequences known as promoters and enhancers, containing short oligonucleotide motifs recognized by trans-factors (TFs) and referred to as cis-active sequences [6,7]. Most biologically relevant cis-active motifs are present within the proximal promoter region, approximately up to 300 bp upstream and 200 bp downstream from the transcription start site (TSS) [8]. However, in in vivo conditions, the interaction between TFs and cis-active sequences is not precisely consistent with the DNA sequences characterized by position weight matrices (PWM). TFs often interact with imperfectly matched cis-active sequences, taking into account the chromatin state and potential of TFs to form dimer/tetramer or oligomer complexes [9]. The occurrence of TFs dimerization/tetramerization process in a homo-or hetero-manner could change such parameters as the affinity of TFs to DNA and their regulatory potential or switch the mode of gene expression regulation from positive to negative or vice versa [10,11]. The formation of such TFs complexes may be supported by the presence of closely spaced tracts of cis-active sequences, concentrated predominantly in evolutionary conservative fragments of promoters known as modules [12]. Ultimately, TFs interact with mediator components and RNA polymerase II to build a multicomponent preinitiation complex, capable of initiating the gene expression process [13].

In the model plant *A. thaliana,* there exist 25,498 genes; among them, about 16.9% encode proteins related to transcription [14]. In other plants, a lower ratio is observed with 5–7% of TFs among all genes, different from the 8–23% representation of *Arabidopsis* proteins involved in transcription in other eukaryotic genomes [14,15]. These TFs are organized in functionally linked and hierarchically structured networks, regulating the expression of other TFs and target genes [16].

Among the included articles, some directly analyze a particular TF or TF family and its role in plant biological processes [17,18,19,20]. Other articles characterize the transcriptional regulation of genes associated with important metabolic processes, such as wax component biosynthesis, phytic acid metabolism, and response to cold or heat stress [21,22,23,24]. One paper describes the structure of the *Arabidopsis* elongator complex, which regulates not only transcription elongation but also the translation process [25]. A review article analyzes the mechanisms leading to NF-Y TFs family expansion in plant genomes [26].

In the article presented by Mendez et al. (2024), a novel MADS-box TF from *Pinus radiata* (*Pr*MADS11) was cloned and functionally characterized [17]. Analysis of the aa sequence revealed the structural features of MIKC type MADS-box TFs. The expression of *Pr*MADS11 could be stimulated by plant vertical loss, where the TF putatively regulates the biosynthesis of cell wall components. The results of transcriptomic studies indicate that *Pr*MADS11 induces the phenylpropanoid route, driving the pathway toward the biosynthesis of lignin precursors known as monolignols and reducing the biosynthesis of anthocyanins [17].

A study on the regulation of 311 genes related to iron metabolism in *Dendrocalamus latiflorus* (sweet bamboo) suggests the predominant role of ERF and DOF TFs [18]. Initial in silico studies showed that the cis-active motifs in IMR gene promoters were most often recognized by the ERF family members, totaling 4821 sites, followed by DOF and BBR-BPC, with 1173 and 1105 binding sites, respectively [18]. However, the highest number of 209 IMR genes was controlled by DOF TFs, while 176 genes were regulated by ERF. The higher concentration of ERF binding sites, compared to those of DOFs, in the IMR genes, indicates a putative stronger influence of ERFs on a smaller subset of genes and reflects a probable bias towards ERFs’ interaction with other TFs [27]. Transcriptomic studies combined with co-expression and RT-PCR analysis confirm IMR genes’ regulation by DOF TFs in *Dendrocalamus latiflorus* [18].

The research by Tiika et al. (2025) analyzed the plant transcriptional response to particular environmental conditions [21]. The authors conducted qRT-PCR studies of genes participating in wax biosynthesis in *Salicornia europaea*, an annual succulent halophyte belonging to the *Amaranthaceae* family. *S. europaea* is one of the most salt-resistant plants worldwide, growing at a 50 to 400 mM NaCl concentration and surviving a treatment up to 1000 mM NaCl [28]. This study contributes to valuable progress in characterizing the mechanisms that enable plant species to grow at increased salt concentrations, as increasing soil salinity is a major environmental factor that adversely affects plant growth and production [29]. Cuticular wax aliphatic compounds consist of a mixture of very long carbon chain molecules, ranging from 22 to 36 carbon atoms [30].

The cuticular wax in *S. europaea* is mainly composed of fatty acids (25.3%), alcohols (46.8%), alkenes (23.9%), and esters (4.1%). Alcohols are the dominant components, with docosanol as the main alcohol compound [21]. The cuticular wax components are generally produced by two biosynthesis branches, i.e., the alcohol-forming pathway, which produces very-long-chain (VLC)-primary alcohols (1° alcohols) and wax esters, and the alkane-forming pathway, which leads to the formation of VLC-aldehydes, VLC-alkanes and their derivatives, secondary alcohols (2° alcohols), and ketones [30].

The authors searched the available databases to identify the following: sixteen wax synthase/diacylglycerol acyltransferases (WS/DGATs), three fatty alcohol oxidases (FAOs) that encode enzymes with aldehyde decarbonylase activity, and eight medium-chain alkane hydroxylases (MAHs) (alkane hydroxylase) that hydroxylate alkanes at the penultimate carbon atom, forming secondary alcohols and ketones [21,30].

The treatment of NaCl enhanced the density of wax crystals, which reached the highest 1.36-fold increase compared to the control after employing 600 mM NaCl [21].

The qRT-PCR analysis of cuticular wax biosynthesis gene expression indicated that genes associated with both branches of wax formation, i.e., the alcohol-forming pathway and the alkane-forming pathway, were stimulated by the 100 and 600 mM NaCl applications [21].

Xu et al. (2024) studied the expression of Nuclear Factor Y A (NF-YA) transcription factors in blueberry (*Vaccinium corymbosum*) [19]. These TFs are involved in multiple plant biological processes such as abiotic stress response, embryogenesis, and abscisic acid signaling.

The blueberry, rich in anthocyanidins, flavonoids, and polyphenols, shows antioxidant properties, which translates into significant medicinal and health-maintaining applications. Although the plant has a high economic and ecological value, its shallow roots make it relatively sensitive to abiotic stresses such as drought, alkali, or salt exposure [31,32].

Analysis of the NF-YA TFs within the whole blueberry genome was possible after the plant genome annotation [33]. The obtained results indicated that most *VcNF-YA* genes exhibited higher expression levels in the stems and leaves, compared to the roots [19]. Moreover, on the basis of in silico analysis of 2 kb-long promoter regions of 24 NF-YA transcription factors, the authors determined their putative responsiveness to light, anaerobic conditions, MeJA, abscisic acid, and drought [19].

An RT-PCR experiment was performed to validate these observations. The results showed that all *VcNF-YA*s were responsive to 100 μM ABA treatment, predominantly indicating increased expression [19]. *VcNF-YA1/2/21*, *VcNF-YA3/5/10*, *VcNF-YA7*, *VcNF-YA8*, *VcNF-YA13/15/18*, and *VcNF-YA17* were upregulated by 200 mM NACl, and *VcNF-YA4/14/20*, *VcNF-YA6*, *VcNF-YA11/23/24*, and *VcNF-YA12/16/19* were downregulated [19].

Blueberry exposure to cold stress (4 °C, 3 h) resulted in the upregulation of *VcNF-YA1/2/21*, *VcNF-YA3/5/10*, *VcNF-YA9*, *VcNF-YA11/23/24*, and *VcNF-YA22*, while the expression trends of *VcNF-YA4/14/20*, *VcNF-YA6*, *VcNF-YA7*, *VcNF-YA8*, *VcNF-YA12/16/19*, *VcNF-YA13/15/18*, and *VcNF-YA17* were downregulated [19].

The study of Yan et al. (2024) analyzed the putative role of *HSP20* genes in maize to elucidate their role in plant heat shock tolerance [22]. The significance of studies on the regulation of maize heat-stress response is due to the plant’s pivotal role in global food supply. It is used not only as a staple food but also in the production of animal feed, biofuels, corn oil, and corn syrup [34,35]. Maize uses the most productive C_4_ mode of photosynthesis in land plants, which results in the high productivity of corn and green biomass [36]. However, global climate warming contains risks for future maize cultivation, as it is estimated that for every 1 °C increase in the global average temperature, the maize yield decreases by 7.4% [37].

Yan et al. (2024) identified and functionally analyzed the *HSP20* gene family in the maize (*Zea mays*) pan-genome to better characterize the plant’s sensitivity to heat stress [22]. Application of the HMM (Hidden Markov Model) search in the maize pan-genome, targeting the conserved HSP20 domain, allowed for the identification of 2430 *HSP20* genes across 57 genomes from 55 maize inbreds or relatives [22].

The authors applied the Ka/Ks values to identify genes showing purifying selection (0 < Ka/Ks < 1) or Ka/Ks > 1, indicating genes under a strong positive selection pressure during maize evolution in some or most inbred lines [22].

The analysis of the available transcriptomic data suggests that changes in the gene expression of *HSP20* genes following heat stress treatment include the majority of Class B and Class D genes, as well as a few Class C and Class A genes. This upregulation was observed in various maize tissues but was more evident in tassels and leaves than in other tissues [22].

Investigation of genes co-expressed with *HSP20* in maize suggests that, in addition to playing a role in the response to heat stress, *HSP20* genes may also be involved in regulating various other biological processes such as secondary metabolism, plant defense mechanisms, and developmental regulation [22]. Further, co-expression and network analysis studies suggest that HSFs could regulate the expression of numerous *HSP20* genes [22].

Another analyzed gene family was the cyclic nucleotide-gated channel (*CNGC*) in luffa (*Luffa cylindrica* L.), encoding a non-selective cation channel, which performs an important role in plant signal transduction [38,39,40]. One study on luffa produced results from the worldwide cultivation of this low-temperature-sensitive vegetable [41]. Similarly to the case of other cucurbit crops, low temperatures before flowering can cause damage, such as flower topping or poor fertilization, negatively affecting the quality and crop yields [42]. The availability of the luffa’s reference genome enabled the genome-wide studies of twenty *CNGC* genes [43].

The results of the cis-active motif distribution within the 2 kb-long promoters of twenty *CNGS*s genes suggest that 50% and 60% of all cis-acting elements in the *LcCNGC* genes were associated with MeJA and ABA signaling, respectively [23].

Additionally, cis-active elements were detected, associated with the response to gibberellins, drought, and salicylic acid. These findings suggest that the *LcCNGC* genes in luffa are multifunctional, probably because they contain corresponding *cis*-acting elements that can be transcriptionally regulated by MYB or WRKY transcription factors, responsive to a variety of stresses including drought and low temperature. Analysis of the available transcriptomic studies suggests that *LcCNGC8 *and *LcCNGC13* showed the highest transcript levels at all time points during the low-temperature treatment, which indicates high responsiveness to cold stress [23].

The pattern of cold response in luffa’s CNGSs genes was confirmed via RNAseq experiments and validated using qRT-PCR [23].

The research presented by Zeng et al. (2024) concentrated on *Dendrocalamus latiflorus*, a clumpy woody bamboo grown in tropical and subtropical regions [20,44]. Although the plant has significant economic value, flowering occurs rarely [45], hampering the seed development and genetic improvement of *D. latiflorus* [46]. The *DlbHLH* TFs were studied as putative regulators of the *D. latiflorus* flowering process [20].

The authors identified 309 *bHLH* TFs in the *D. latiflorus* genome [20]. To understand the significance of the *bHLH* gene family in regulating the flower development of *D. latiflorus*, the *bHLH* gene family was identified and analyzed in this study. The phylogenetic analysis of the mutation rates between Ka (nonsynonymous) and Ks (synonymous) allowed the calculation of the selection pressure of gene duplications. *DlbHLH* gene pairs underwent purifying selection during evolution, as suggested by Ka/Ks ratios below 1, with the dominant rate under 0.5 [20]. Analysis of the promoter regions of *DlbHLH* genes revealed the important role of light, phytohormones, and stress factors in gene expression regulation. The role of *DlbHLH* at different stages of floral organ development was elucidated via the analysis of available transcriptomic data. As a result, a group of *DlbHLH* genes playing stage-specific roles in flower development were identified [20]. The obtained results, validated via RT-PCR experiment, were consistent with the RNAseq data. *DlbHLH95*, *DlbHLH123,* and *DlbHLH264* were highly expressed in late flower development stages, while *DlbHLH97*, *DlbHLH118,* and *DlbHLH165* were significantly highly expressed in the F1 stage. Moreover, *DlbHLH135* and *DlbHLH150* were highly expressed in all four flower developmental stages [20].

The findings obtained by Peng et al. (2024) suggest that in *A. thaliana,* the plant senescence induced by ethylene is regulated by multiple inositol phosphate phosphatase (*At*MINPP) genes [24]. The overexpression of *AtMINPP* (*AtMINPP–OE*) results in an increased expression of senescence-associated genes such as *SAG12*, *SAG13*, *SAG29*, *SAG113*, *SAG201*, *ANAC047*, *SENESCENCE1* (*SEN1*), *SEN4, SENESCENCE–INDUCED RECEPTOR–LIKE KINASE* (*SIRK*), and *BFN1*. The phenotype outcome was early leaf senescence and reduced chlorophyll content [24]. Following application of known ethylene biosynthesis inhibitors, such as AgNO_3_ and aminoethoxyvinylglycine (AVG), to *AtMINPP–OE* mutants, the genetic and phenotype changes reversed [24].

The loss-of-function heterozygous mutant (*atminpp/+*) showed the opposite genetic changes and phenotype. The yeast one-hybrid and chromatin immunoprecipitation assays indicated that the ethylene-responsive EIN3 transcription factor directly binds to the promoter of *AtMINPP.* The genetic relationship between EIN3 and *At*MINPP in controlling leaf senescence was analyzed in a double-mutant *ein3–1eil1–3* overexpressing *AtMINPP* [24]. The *AtMINPP–OE* significantly augmented leaf senescence in the *ein3–1eil1–3* mutant background [24].

Several papers analyzed gene expression patterns in the context of the interaction between the cis-active motif and corresponding TF before the start of transcription [17,18,19,20,21,22]. The later stage of gene expression, i.e., transcription elongation, was analyzed in the work of Jun et al. (2024) [25]. In yeast, the elongator complex consists of six subunits (Elp1–Elp6), where only one Elp3 (ScElp3) indicates acetyltransferase, contributing to protein and, importantly, to tRNA modification [47,48,49,50,51]. Involvement of the elongator complex in tRNA modification in plants is responsible for its pivotal role in mRNA translation elongation. Jun et al. (2024) studied the protein–protein interactions between *Arabidopsis* ELP4, ELP5, and ELP6 proteins [25]. The results of the Y2H screening showed that *At*ELP4 interacted with AtELP6 but not directly with *At*ELP5. The collected data suggest that *Arabidopsis* ELP4, ELP5, and ELP6 proteins form a heterotrimer, with ELP6 serving as a bridge [25]. Furthermore, the authors observed that the *Arabidopsis* elongator-associated protein, Deformed Roots and Leaves 1 (DRL1), does not directly interact with *At*ELP proteins [25]. Despite the relatively low sequence homology, the structure of the ELP456 sub-complex in *Arabidopsis* and yeast is similar, particularly within the RecA-ATPase fold and the localization of hydrogen bonds [25].

The review article of Siriwardana (2025) analyzed the mechanisms of gene duplication and retention of plant NF-Y TFs, which could be used as a model in other plant gene families [26]. The NF-Y, also called the CCAAT-binding factor (CBF) or heme activator protein (HAP), is a transcription factor family found in all eukaryotes. It plays an essential role in eukaryotes, as the loss of function of subunits is lethal to embryos [52]. The NF-Y TFs is a trimeric complex composed of three subunits, i.e., NF-YA, NF-YB, and NF-YC, comprising three independent protein families: NF-YA, NF-YB, and NF-YC [53,54].

The frequency of gene duplication and retention is higher in plants than other eukaryotes, resulting in a more common polyploidy and a much broader variation in genome size. The plant genome size ranges from ~63 Mb to 150 Gb in dicots [55], contrasting with the variation in genome size from ~1.6 Gb to 8 Gb in animals [55].

Although the most common fate of duplicated genes is loss [56], genetic redundancy is a common feature in the genomes of higher organisms [57]. Among numerous mechanisms leading to duplicated gene retention, a prominent role is played by subfunctionalization and neofunctionalization. Subfunctionalization occurs when each daughter copy of the duplicated gene indicates part of the function of the parental gene [58]. In neofunctionalization, one gene retains the ancestral function, whereas the paralog assumes a novel one [58].

The presented results suggest that NF-YA, NF-YB, and NF-YC family members could be classified into ancestrally related subclasses, allowing for studies on the mechanisms that lead to gene retention after duplication. Analysis of multiple sequence alignments (MSAs) of NF-Y suggests the presence of a single highly conserved core domain flanked by non-conserved N- and C-termini within NF-YA, NF-YB, and NF-YC [26].

The conserved core HFD domain may be under pressure for purifying selections (negative), while the N- and C-terminal segments may be under pressure for diversifying (positive) selection. The NF-Y THs could be used as a putative model for future studies on the expansion of gene families in plants [26].
